# Low-level laser therapy: Case-control study in dogs with sterile pyogranulomatous pododermatitis

**DOI:** 10.14202/vetworld.2016.882-887

**Published:** 2016-08-22

**Authors:** Roberta Perego, D. Proverbio, A. Zuccaro, E. Spada

**Affiliations:** Department of Veterinary Sciences for Health, Animal Production and Food Safety (VESPA), University of Milan, Via Celoria 10, 20133 Milano, Italy

**Keywords:** case-control study, dog, low-level laser therapy, skin, sterile pyogranulomatous pododermatitis

## Abstract

**Aim:**

Low-level laser therapy (LLLT) is a therapeutic photobiostimulation with properties in reducing swelling, inflammation, and promoting tissue healing. The objective of this pilot study was to evaluate LLLT in sterile pyogranulomatous pododermatitis in five dogs.

**Materials and Methods:**

In each dog, one lesion was designated as the control (treated with a 0.0584% hydrocortisone aceponate spray), and one or more other lesions were treated with a gallium aluminum arsenide-laser, daily for 5 days. Lesions were scored before treatment (D0), at the end (D4), 16 days after the last laser treatment (D20), and after 2 months (D65).

**Results:**

Comparing the treated lesion group with the control lesion group, the clinical score was similar at D0, whereas there was a statistically significant difference at D4 and D20; in the treated group over time, there was a statistically significant improvement between D0, D4, and D20. Lesion recurrence was absent in more than 50% of the treated lesions at D65. No adverse reactions were reported.

**Conclusion:**

Given the positive results of this first clinical study, it would be interesting to extend the study to confirm the validity of this type of therapy in sterile pyogranulomatous pododermatitis in the dog.

## Introduction

Low-level laser therapy (LLLT) is a therapeutic modality of photobiostimulation that uses the emission of a coherent (laser) or non-coherent (filtered lamps or light emitting diode [LED]) light source, or a combination of both [1]. It uses red and near-infrared (NIR) light with wavelengths between 300 and 10,600 nm. Various substrates, including more recently gallium aluminum arsenide (GaAlAs), have been used to create the lasers used for LLLT [2].

The basic biological mechanisms responsible for the effects of LLLT are not yet fully understood [1]. The effects are thought to be mediated by absorption of red and NIR light by mitochondrial chromophores, in particular, cytochrome C oxidase [3]. Consequently, a cascade of events occur, with upregulation of oxidative phosphorylation and increased production of adenosine triphosphate, leading to activation of fibroblasts, keratinocytes, and endothelial cells, producing a reduction of pain, edema, and inflammation [4].

Although most of the aforementioned *in vitro* studies appear to show significant effects in a number of tissues after exposure to LLLT, published clinical studies, particularly in dermatology, have shown conflicting results. In human medicine, some studies have reported successes with LLLT in the treatment of ulcers [5], keloids [6], and skin wounds [7]. However, there are other studies [8,9] that have shown LLLT to be ineffective in promoting the healing of skin wounds and ulcers.

Similar limitations are also found in animal models: Some studies show a significant acceleration of the healing of experimentally induced wounds or burns in rats or mice treated with LLLT [10,11], but many studies in the same species and also in porcine models [12] have failed to show any positive effects. The main problem appears to be the absence of a standard protocol, and the difficulty in determining the wavelengths, the power, the energy density, and the medium most appropriate for the different therapeutic treatments [1,7].

There are a few clinical veterinary studies that have evaluated the efficacy of LLLT on the healing of skin wounds: In the horse, with unsuccessful outcomes [13,14], in cattle [15] and only few studies in the dog, among which a clinical study on a individual patient with a favorable outcome [16] and a experimental study on five male beagle dogs, with no apparent beneficial effects [17].

Sterile pyogranulomatous pododermatitis is a disease with unclear etiopathogenesis that affects the pedal skin of dogs [18]. Clinical signs usually consist of single or multiple nodules that may or may not be accompanied by draining sinus tracts [19]. Histopathological examination shows pyogranulomatous dermatitis, although bacterial and fungal cultures of lesional material are negative [18]. A diagnosis of sterile pyogranulomatous pododermatitis carries a good to fair prognosis. Glucocorticoids, tetracycline, and niacinamide or other immunomodulatory agents such as cyclosporine or azathioprine are necessary to control clinical signs [20,21].

Given the reported properties of LLLT in reducing pain, swelling and inflammation and promoting tissue healing, and the complexity of the prolonged therapeutic management of sterile pyogranulomatous pododermatitis, the purpose of this clinical study was to evaluate the effects of LLLT treatment in dogs with idiopathic sterile (pyo)granulomatous pododermatitis.

## Materials and Methods

### Ethical approval

This study was performed at the Department of Veterinary Sciences for Health, Animal Production and Food Safety (VESPA) of the University of Milan, and it did not include experimental animals, and it was performed on client-owned dogs referred to our institution for therapeutic purpose. All owners gave informed consent for treatments, measurements, and for data recording. The study was carried out in accordance with Italian law (DL 14^th^ March 2014 n.26) and Europe Union legislation covering the use of animal for the scientific purpose (“Animal Scientific Procedures Act” 63/2010/EU) and with the Institutional Ethical Guidelines.

### Inclusion criteria and clinical procedures

Client-owned dogs prospectively included in the study were presented at the dermatological ambulatory of the Department of Veterinary Sciences for Health, Animal Production and Food Safety (VESPA) of the University of Milan, from January 2013 to December 2014. Animals were deemed eligible for enrollment once they satisfied a series of inclusion criteria: (i) The presence of pedal lesions for >4 months and the absence of cutaneous lesions at other body sites; (ii) diagnosis of sterile pyogranulomatous pododermatitis according to the criteria in the literature [18]. Briefly, results of skin scrapings/hair plucks, cytology, fungal culture, microbial culture (biopsy specimens) for aerobic, anaerobic bacteria and mycobacteria, intradermal testing, food elimination trial, hematology, biochemistry, urinalysis, *Leishmania infantum* indirect fluorescent antibody test and polymerase chain reaction, thyroid stimulating hormone and free T_4_ evaluation, histopathology and immunohistochemical staining of multiple lesional biopsies failed to yield a specific etiological diagnosis. Dermatoistophatology only revealed nodular to diffuse, granulomatous to pyogranulomatous dermatitis; (iii) have at least two active nodular lesions present in different limbs, which were similar in size and clinical score (see above) at the day of inclusion (one as a control and the other/s to receive laser therapy); (iv) have previously subjected to local or systemic immunosuppressive drugs and/or courses of antibiotic therapy with only temporary or partial remission of the disease; (v) have not received any topical or systemic medication in the 15 days preceding the study.

On the day of the inclusion (D0), signalment and history were recorded and a complete physical examination was performed. Each patient was classified as cooperative/non-cooperative according to whether they would sit still for at least 10 min on the examination table. Only cooperative dogs were included in the study. Dogs with a history of neoplastic disease and pregnant bitches were excluded as recommended by the manufacturer of the laser device. A complete dermatological examination was then performed, the interdigital lesions and their location were recorded, and multiple cytological samples from each lesion examined to rule out concomitant bacterial or fungal infection. Each dog was, therefore, both a “treated” and a “control.” The selection of lesion/s to be treated, and the one to use as a control, was done in a random, blind fashion by an operator not involved in the study. A clinical score was calculated for each lesion (Table-1), at D0, at subsequent daily sessions of LLLT (D1, D2, D3, and D4) and follow-up 16 days after the final session of LLLT (D20), always in blind fashion by an operator not involved in the study. The scoring system used was derived (with appropriate changes and addictions relating to the nature of the lesions in this study) from the Pressure Ulcer Scale for Healing which had been developed by the National Pressure Ulcer Advisory Panel as a tool to monitor changes in pressure ulcers over time in human patients [22], the Bates-Jensen Wound Assessment Tool [23], and the wound bed scoring for chronic wounds [24].

**Table-1:** Clinical score for the objective assessment of lesions derived and modified from PUSH [22], BWAT [23], and WBS [24] scales.

Parameters	0	1	2	3	4
Skin color	Pink	Erythematous	Dark	-	-
Pain	None	Present	-	-	-
Granulation tissue	None	Present	-	-	-
Exudate (type)	None	Serous	Serohemorrhagic	Hemorrhagic	Purulent
Exudate (amount)	None	Light	Moderate	Heavy	-
Eschar	None	Present 025% surface area	Present, >25% surface area	-	-
Fistula	None	Present	-	-	-
Lesion size (diameter in cm)	0	<0.5	0.51	13	>3

PUSH=Pressure ulcer scale for healing, BWAT=Bates-Jensen wound assessment tool, WBS=Wound bed scoring

All lesions were photographed at D0 and each session of laser therapy/next visit. No other medications except that provided by the study for the control lesions were permitted during the study period.

The laser used for this study was the B-808-CURE LLLT (Good-Energies), a portable laser for human use, lightweight (173 g), and equipped with rechargeable batteries. It is a diode laser having a GaAlAs solid medium, with a power of 250 mW (micro-pulsed to ensure greater effectiveness and penetration) and a wavelength of 808 nm (infrared). The energy density is 0.9 J/min/cm^2^, whereas the peak energy of 14.4 J/min is for the entire treated surface.

An important feature of this instrument is that the rectangular area of irradiation surface is visible due to the presence of a green LED, of 4.5 cm². This allows the operator to apply therapy over a wider area while maintaining a constant coherence of the beam at each point of the treated area.

The dogs included in the study received the following treatment protocol on the control lesion:

- From D0 to D4 application once a day of 0.0584% hydrocortisone aceponate (HCA) spray (Cortavance^®^; Virbac SA)
- D20: Follow-up 16 days after the last spray application.

The same dogs included in the study received the following treatment protocol on the lesion/s to be treated:

- D0: Initial application of LLLT, maintaining the device as close as possible to the lesion without touching, for 1.5 min to record any side effects followed by the second session of 6 min after an interval of at least 30 min
- D1, D2, D3, and D4: 1 daily treatment with a duration of 6 min
- D20: Follow-up 16 days after the last laser application.

The control and treated lesions were not been subject to any pre-treatment (such as cleaning or clipping the surface) before HCA spray or LLLT therapy.

The dogs were subsequently re-evaluated in an indirect manner through telephone interviews with their owners 2 months after the last application of LLLT (D65), to assess the progression of both treated and control lesions and the appearance of any similar new lesions.

This protocol was selected following the therapeutic indications suggested by the manufacturer of the laser device in their guidelines for human cases of “wound healing” and “fresh and old wounds and surgical scars.” The control lesions did not receive any treatment.

### Statistical analysis

The analysis of the results was carried out using statistical software MedCalc (version 12.7.8.0). The total population of treated and controls was assessed by the Kolmogorov-Smirnov test which showed a normal distribution of available values. A paired t-test was used to compare scores in treated lesions at D0, D1, D2, D3, D4, and D20. The same evaluation was performed in the control group lesions. The un-paired t-test was used for comparison of the scores of the treated group and the control group at the three stages of assessment D0, D4, and D20. For all statistical tests, the threshold of significance was set at p<0.05.

## Results

Nine dogs were enrolled in the study, but 3 dogs were lost during the LLLT between D2 and D4 and 1 dog not come to the follow-up visit after 16 days. Then, 5 dogs were finally included in the study (for a total of 7 treated lesions), 2 females and 3 males, aged between 2 and 9 years old, of 3 different breeds (1 crossbreed, 1 Cane Corso, 3 English Bulldog). Table-2 summarizes the location of the 5 control lesion (C) and the 7 lesion/s subjected to laser treatment (T1 and T2) in each dog, whereas Table-3 shows the detailed clinical score for each parameter measured, and then, the total score for each dog, for both control lesion and treated lesion/s, at D0, D4, and D20.

**Table-2:** Site of the interdigital lesion used as a control and lesion/s receiving laser therapy for each dog in the study.

Dog number	Control lesion	First treated lesion	Second treated lesion
01	Left foreleg III-IV digit	Right foreleg III-IV digit	Left hind limb III-IV digit
02	Right foreleg III-IV digit	Left foreleg II-III digit	Left hind limb III-IV digit
03	Right foreleg III-IV digit	Left foreleg IVV digit	-
04	Left foreleg III-IV digit	Right foreleg IVV digit	-
05	Right foreleg III-IV digit	Left hind limb III-IV digit	-

**Table-3:** Total clinical scores for each dog of 7 treated lesions (T1 and T2) and 5 control lesions (C), on the first (D0) and the last day of treatment (D4) and at followup (D20).

Dog number	Days	Total score		

		T1 (first treated lesion on all 5 subjects)	T2 (second treated lesions only 2 subjects)	C (control lesions)
1	D0	5	4	5
	D4	1	0	4
	D20	1	0	5
2	D0	7	6	6
	D4	0	0	2
	D20	0	0	4
3	D0	9	-	4
	D4	5	-	6
	D20	0	-	6
4	D0	8	-	8
	D4	0	-	2
	D20	2	-	4
5	D0	8	-	8
	D4	2	-	5
	D20	0	-	6

There was no a significant difference in the total clinical score of the treated and control lesions at D0 (p=0.4639) while statistically significant difference was observed at both D4 (p=0.0231) or D20 (p<0.0001). There was a statistically significant difference in the total score of treated lesions over time in the 5 subjects between D0 and D4 (p=0.0001), as well as between D0 and D20 (p=0.0002), but no statistically significant difference was seen between D4 and D20 (p=0.4262). In the control group, there was no statistically significant difference between D0 and D4 (p=0.051) or between D0 and D20 (p=0.1723), but a significant difference was present between D4 and D20 (p=0.0041).

At the telephone interview, 2 months after the last laser treatment (D65), in two subjects, there had been complete resolution of treated lesions with no recurrence while the control lesions remained unchanged; in 1 dog, the treated lesions, although still present, had remained stable while control lesion had progressed; in 1 dog, the treated lesions had not progressed while the control lesion had worsened and new lesions had also appeared in different locations that before; and in the last dog, the treated lesions had completely relapsed within a few weeks of treatment while the control lesion improved over time.

No adverse reactions or local or systemic side effects were reported during or after therapy.

## Discussion

To the authors’ knowledge, this is the first clinical study on the application of LLLT in sterile pyogranulomatous pododermatitis in the dog and one of the first clinical studies on the use of LLLT in canine dermatology.

One recent study on the clinical efficacy of LLLT on localized canine atopic dermatitis shows that the LLLT is not an effective localized treatment for pedal pruritus [25]. The only two other clinical studies in dogs are that by Lucroy *et al*. [16], on a single subject, for the treatment of a chronic non-surgical wound and that by Olivieri *et al*. [26] for the treatment of non-inflammatory alopecia in seven dogs, both with a favorable outcome. It is, therefore, very difficult to compare our data with those reported in the literature, where veterinary studies are almost exclusively animal models for human medicine.

The results of our study of 5 dogs, with a total of 7 treated lesions, seem to indicate a possible positive effect of LLLT in the regression of treated lesions. In fact, there was a decrease in clinical score of treated lesions between the beginning and end of therapy and the beginning of therapy and follow-up. This confirms the trend toward the healing of injuries treated in these five subjects.

When individual clinical parameters are studied, it can be seen that the biggest improvements occurred in type and amount of exudate and the size of the lesion.

The reduction in size of a sterile granulomatous lesion would seem to highlight the role of LLLT in the modulation of the inflammatory process and edema as already demonstrated by other veterinary studies [27]. The reported reduction in exudate in this study is in line with what was observed in the study of Petersen *et al*. [13] on the horse. A reduction of exudation was also demonstrated in a study carried out on a group of mice treated with LLLT after the induction of pleurisy [28].

No statistically significant difference was found in clinical score between the end of laser treatment and the follow-up after 16 days in any treated lesion. This could suggest a sustained effect of LLLT over time and also corroborated by the owners’ reports at 2 months of follow-up (D65) that in more than 50% of the subjects the treated lesions had not recurred or had remained stable. The lasting effect of LLLT could be due to improvements in local pedal microcirculation, as found in the study of Schindl *et al*. [29] on diabetic mice; a factor that seems to be involved in the pathogenesis of foot lesions in dogs [18].

The positive effects of LLLT are corroborated by the analysis of the clinical score in control lesions, where no significant difference was measured in the total score between the start and end of therapy, and between the initial examination and follow-up, whereas between the end of therapy and follow-up the total score increased, with clinical worsening of the lesions. This worsening was confirmed after 2 months through the telephone interview in 2 subjects.

In our study, we chose to use LLLT on dogs with sterile pyogranulomatous pododermatitis, as this represents an uncommon chronic progressive dermatological disease in dogs, that has been documented by case reports and review articles [18,20,30], although many questions remain unanswered about its etiology [18]. Various theories have been proposed to explain the underlying pathogenesis including pedal conformation, trauma, immunosuppression, bacterial infection, furunculosis with dermal granuloma formation [18], and rupture of follicular cysts on ventral interdigital skin, which cause inflammation and bacterial infection and form fistulas that drain onto the dorsal interdigital surface [31]. Some authors have reported that reactions directed against ruptured follicular contents and in-grown hairs are a pivotal driving force in perpetuating the inflammatory response [30]. The term lymphocytic-plasmacytic pododermatitis (LPP) has previously been proposed in a study with a population characterized histopathologically by epidermal hyperplasia, hyperkeratosis, spongiosis, dermal edema and perivascular aggregates of lymphocytes and plasma cells [20].

Sterile pododermatitis can be managed over time with steroid or azathioprine, cyclosporine therapy to control clinical signs [21], and in some cases treated with prolonged antimicrobial therapy and self-vaccination. A surgical fusion podoplasty has been recommended for cases of chronic fibrosing interdigital pyoderma that fail to respond to conventional therapy, but a significant amount of effort is needed in the on-going wound management [18].

In our study, all subjects had previously received courses of antibiotic therapy and local or systemic immunosuppressive drugs with temporary resolution of the disease. The improvements achieved on treated lesions may suggest an important role of LLLT in the treatment of sterile pyogranulomatous pododermatitis. The costs and side effects that would occur as a result of continuous cycles of pharmacological therapy may well be reduced. In the light of the increasing importance of antibiotic resistance, a careful antibiotic stewardship should also be a good reason to explore alternative treatment options for this complicated disease.

Moreover, LLLT may represent a less invasive alternative to surgical therapy for relapsed multiple focal lesions.

However, successful LLLT treatment requires good owner compliance as they must be willing to present their dog for at least one session of laser therapy daily for 5 consecutive days. This treatment protocol can be quite challenging for the owner. In the absence of standard published protocols for LLLT treatment in the dog, it would be useful to test protocols characterized by less frequent outpatient sessions and with increased irradiation time or to perform daily therapy with a pulse manner.

Given that the equipment used in our study is easy to use and to buy for private use and adverse reactions appear uncommon also in dogs, it might be possible to rent the unit directly to the owner who, after a few demonstrations, could manage the daily treatment at home.

The patient’s cooperation during the sessions of laser therapy was important to success in our study. All subjects included in this study were cooperative during the 6 min of treatment, but this reinforced the minimal invasiveness of the instrument. In the study, we reduced treatment frequency to a single daily treatment, instead of the two recommended by the manufacturer for human patients for wound healing, to maximize cooperation of the dog owners. This modification of the recommended human protocol could be considered as a limitation of our study, together with the small number of treated lesions.

Finally, we designed our study as a pilot study of a clinical innovative therapy on a small number of subjects following the literature on some similar studies on new treatments in dermatology [26,32,33]. We preferred to use the “case-control” study design on the same subject to reduce the variables of clinical response and avoid confounding factors such as age, race, and gender. Having an untreated control lesion on the same treated subject allowed us to assess the actual benefit of the LLLT in a disease characterized by spontaneous remission and recrudescence phases considering also the low number of recruited subjects.

Given the positive results of this first clinical study, it would be interesting to extend the study to confirm the validity of this type of therapy in sterile pyogranulomatous pododermatitis in the dog. Since the literature does not contain unique therapeutic protocols for the use of LLLT in most fields of application, either in human or veterinary medicine, the authors believe that further clinical studies are necessary to determine optimal irradiation times, the frequency, and the duration of treatment for dermatological application of low-intensity laser therapy in dogs.

## Conclusion

Given the positive results of this first clinical study, it would be interesting to extend the number of dogs treated to confirm the validity of low level laser therapy in sterile pyogranulomatous pododermatitis in the dog.

## Authors’ Contributions

RP, DP, and ES designed the study. The experiment was done by AZ, DP, and RP. All the authors participated in data analysis, draft, and revision of the manuscript. All authors read and approved the final manuscript.
